# Supplementary material for: Shared decision making applied to self-management program for hypertensive patients: A scoping review protocol
Source: PLoS One. 2024 Nov 21;19(11):e0309593. doi: 10.1371/journal.pone.0309593 (PMC11581201; doi:10.1371/journal.pone.0309593)
Supplement: S1 File — (DOCX) [file pone.0309593.s002.docx]

**S3. Search strategy for MEDLINE (PubMed).**

| No. | Query | Results |
| --- | --- | --- |
| 1 | shared decision*[Title/Abstract] OR sharing decision*[Title/Abstract] OR informed decision[Title/Abstract] OR informed choice*[Title/Abstract] OR decision* aid*[Title/Abstract] | 23,112 |
| 2 | ((share*[Title/Abstract] OR sharing[Title/Abstract] OR informed[Title/Abstract] OR participate*[Title/Abstract] OR support*[Title/Abstract]) AND (decision*[Title/Abstract] OR decid*[Title/Abstract] OR choice*[Title/Abstract])) | 196,809 |
| 3 | #1 OR #2 | 198,287 |
| 4 | "decision making"[MeSH Major Topic] | 103,865 |
| 5 | "decision support techniques"[MeSH Major Topic] | 28,331 |
| 6 | "decision support systems, clinical"[MeSH Major Topic] | 6,814 |
| 7 | "choice behavior"[MeSH Major Topic] | 32,671 |
| 8 | ("decision*"[Title/Abstract] OR "choice*"[Title/Abstract]) AND ("making"[Title/Abstract] OR "support*"[Title/Abstract] OR "behavior*"[Title/Abstract] OR "behaviour*"[Title/Abstract]) | 336,053 |
| 9 | #4 OR #5 OR #6 OR #7 OR #8 | 426,587 |
| 10 | "patient participation"[MeSH Major Topic] | 16,468 |
| 11 | ("patient*"[Title/Abstract] OR "consumer*"[Title/Abstract]) AND ("involv*"[Title/Abstract] OR "participat*"[Title/Abstract] OR "empower*"[Title/Abstract] OR "engage*"[Title/Abstract] OR "partner*"[Title/Abstract]) | 986,631 |
| 12 | #10 OR #11 | 995,940 |
| 13 | "professional patient relations"[MeSH Major Topic] | 71,553 |
| 14 | "nurse*"[Title/Abstract] OR "physician*"[Title/Abstract] OR "clinician*"[Title/Abstract] OR "doctor*"[Title/Abstract] OR "general practitioner*"[Title/Abstract] OR "gp"[Title/Abstract] OR "health care professional*"[Title/Abstract] OR "healthcare professional*"[Title/Abstract] OR "health care provider*"[Title/Abstract] OR "healthcare provider*"[Title/Abstract] OR "resident*"[Title/Abstract] | 1,431,320 |
| 15 | "patient*"[Title/Abstract] OR "consumer*"[Title/Abstract] OR "people*"[Title/Abstract] OR "individual*"[Title/Abstract] | 9,379,902 |
| 16 | #13 OR (#14 AND #15) | 855,312 |
| 17 | "patient centered care"[MeSH Major Topic] | 14,571 |
| 18 | (("patient*"[Title/Abstract] OR "person*"[Title/Abstract] OR "client*"[Title/Abstract] OR "consumer*"[All Fields]) AND "NEAR"[All Fields]) AND ("centred"[Title/Abstract] OR "centered"[Title/Abstract] OR "focused"[Title/Abstract] OR "oriented"[Title/Abstract]) | 2,124 |
| 19 | #17 OR #18 | 16,670 |
| 20 | #9 AND #12 | 41,137 |
| 21 | #9 AND #16 | 65,548 |
| 22 | #3 OR #20 OR #21 OR #19 | 252,996 |
| 23 | "hypertension"[MeSH Major Topic] | 230,977 |
| 24 | "hypertens*"[Text Word] OR "antihypertens*"[Text Word] | 596,956 |
| 25 | ("high"[Text Word] OR "elevat*"[Text Word] OR "rais*"[Text Word]) AND "blood pressure"[Text Word] | 141,677 |
| 26 | #23 OR #24 OR #25 OR #26 | 670,767 |
| 27 | "self care"[MeSH Major Topic] | 27,720 |
| 28 | "educational status"[MeSH Major Topic] OR "education"[MeSH Major Topic] | 573,094 |
| 29 | "patient education as topic"[MeSH Major Topic] | 41,094 |
| 30 | "educat*"[Title/Abstract] OR "self manag*"[Title/Abstract] OR "self manag*"[Title/Abstract] OR "self car*"[Title/Abstract] OR "self car*"[Title/Abstract] OR "train*"[Title/Abstract] OR "instruct*"[Title/Abstract] OR "patient education"[Title/Abstract] OR "management-plan"[Title/Abstract] OR "management-plan"[Title/Abstract] OR (("management*"[All Fields] AND "NEAR1"[All Fields]) AND "program*"[Title/Abstract]) OR (("disease*"[All Fields] AND "NEAR2"[All Fields]) AND "management*"[Title/Abstract]) | 1,399,312 |
| 31 | #27 OR #28 OR #29 OR #30 | 1,692,518 |
| 32 | #22 AND #26 AND #31 | 770 |
