# Supplementary material for: Shared decision making applied to self-management program for hypertensive patients: A scoping review protocol
Source: PLoS One. 2024 Nov 21;19(11):e0309593. doi: 10.1371/journal.pone.0309593 (PMC11581201; doi:10.1371/journal.pone.0309593)
Supplement: Attachment — Submitted filename: PONE-D-23-31258 reviewed.docx [file pone.0309593.s003.docx]

PLOS ONE

Shared decision making applied to self-management program for hypertensive patients: A scoping review protocol

--Manuscript Draft--

| Manuscript Number: | PONE-D-23-31258 |
| --- | --- |
| Article Type: | Study Protocol |
| Full Title: | Shared decision making applied to self-management program for hypertensive patients: A scoping review protocol |
| Short Title: | Shared decision making applied to self-management program scoping review protocol |
| Corresponding Author: | Mi Ok Song Nambu University  Gwangju, Jeolanamdo KOREA, REPUBLIC OF |
| Keywords: | Decision making, Shared; self-management; hypertension; Scoping Review |
| Abstract: | Introduction: Shared decision making (SDM) improves health outcomes by providing individualized nursing interventions to patients and educating and empowering them to actively participate in health. However, hypertensive patients who require self- management do not actively participate in the SDM process.  Aims: This scoping review aims to investigate the available information on SDM to self- management programs for hypertensive patients.  Methodology: The proposed scoping review protocol will be conducted using the Arksey and O’Malley methodological framework, following the Joanna Briggs Institute’s methodology for scoping reviews. Multiple databases will be searched, including the MEDLINE (PubMed), Embase, Cochrane Library and Google Scholar. Papers’ titles, abstracts, and full text will predominantly be screened by one researcher with a second researcher. Data will be extracted using a customized table developed in collaboration with the research team, and presented in tabular format, arranged thematically. In the scoping review, studies involving the self- management program applied for hypertensive patients with two or more of the components of the intervention are included. And in the SDM process, studies in which healthcare providers provide various options for patients' self- management and set health goals in consideration of patients' preferences and values will be included.  Registration: The scoping review protocol has been registered on the Open Science Framework (OSF) repository Registries (https://osf.io/rtfvq;  DOI: 10.17605/OSF.IO/D8SPM). |
| Order of Authors: | So Young Yun |
|  | Mi Ok Song |
| Additional Information: |  |
| Question | Response |
| Financial Disclosure  Enter a financial disclosure statement that describes the sources of funding for the work included in this submission. Review the [submission guidelines](http://journals.plos.org/plosone/s/submission-guidelines#loc-financial-disclosure-statement) for detailed requirements. View published research articles from [*PLOS ONE*](https://journals.plos.org/plosone/) for specific examples.  This statement is required for submission and will appear in the published article if the submission is accepted. Please make | Yes |

*Powered by Editorial Manager® and ProduXion Manager® from Aries Systems Corporation*

| sure it is accurate. |  |
| --- | --- |
| Funded studies  Enter a statement with the following details:   - Initials of the authors who received each award - Grant numbers awarded to each author - The full name of each funder - URL of each funder website - Did the sponsors or funders play any role in the study design, data collection and analysis, decision to publish, or preparation of the manuscript? |  |
| Did you receive funding for this work? |  |
| Please add funding details.  as follow-up to "Financial Disclosure  Enter a financial disclosure statement that describes the sources of funding for the work included in this submission. Review the [submission guidelines](http://journals.plos.org/plosone/s/submission-guidelines#loc-financial-disclosure-statement) for detailed requirements. View published research articles from [*PLOS ONE*](https://journals.plos.org/plosone/) for specific examples.  This statement is required for submission and will appear in the published article if the submission is accepted. Please make sure it is accurate. | This study was supported by research funds from Nambu University, 2022 |
| Funded studies  Enter a statement with the following details:   - Initials of the authors who received each award - Grant numbers awarded to each author - The full name of each funder - URL of each funder website - Did the sponsors or funders play any role in the study design, data collection and analysis, decision to publish, or preparation of the manuscript? |  |
| Did you receive funding for this work?" |  |
| Please select the country of your main research funder (please select carefully | KOREA, REPUBLIC OF - KR |

*Powered by Editorial Manager® and ProduXion Manager® from Aries Systems Corporation*

| as in some cases this is used in fee calculation).  as follow-up to "Financial Disclosure  Enter a financial disclosure statement that describes the sources of funding for the work included in this submission. Review the [submission guidelines](http://journals.plos.org/plosone/s/submission-guidelines#loc-financial-disclosure-statement) for detailed requirements. View published research articles from [*PLOS ONE*](https://journals.plos.org/plosone/) for specific examples.  This statement is required for submission and will appear in the published article if the submission is accepted. Please make sure it is accurate. |  |
| --- | --- |
| Funded studies  Enter a statement with the following details:   - Initials of the authors who received each award - Grant numbers awarded to each author - The full name of each funder - URL of each funder website - Did the sponsors or funders play any role in the study design, data collection and analysis, decision to publish, or preparation of the manuscript? |  |
| Did you receive funding for this work?" |  |
| Competing Interests  Use the instructions below to enter a competing interest statement for this submission. On behalf of all authors, disclose any [competing interests](http://journals.plos.org/plosone/s/competing-interests) that could be perceived to bias this work—acknowledging all financial support and any other relevant financial or non- financial competing interests.  This statement is required for submission and will appear in the published article if the submission is accepted. Please make sure it is accurate and that any funding sources listed in your Funding Information later in the submission form are also | The authors have declared that no competing interests exist. |

*Powered by Editorial Manager® and ProduXion Manager® from Aries Systems Corporation*

| declared in your Financial Disclosure statement.  View published research articles from  [*PLOS ONE*](http://journals.plos.org/plosone/) for specific examples. |  |
| --- | --- |
| NO authors have competing interests  Enter: *The authors have declared that no competing interests exist.*  Authors with competing interests  Enter competing interest details beginning with this statement:  *I have read the journal's policy and the authors of this manuscript have the following competing interests: [insert competing interests here]* |  |
| * typeset |  |
| Ethics Statement  Enter an ethics statement for this submission. This statement is required if the study involved:   - Human participants - Human specimens or tissue - Vertebrate animals or cephalopods - Vertebrate embryos or tissues - Field research   Write "N/A" if the submission does not require an ethics statement.  General guidance is provided below. Consult the [submission guidelines](http://journals.plos.org/plosone/s/submission-guidelines#loc-guidelines-for-specific-study-types) for detailed instructions. Make sure that all information entered here is included in the Methods section of the manuscript. | N/A |

*Powered by Editorial Manager® and ProduXion Manager® from Aries Systems Corporation*

| Format for specific study types  Human Subject Research (involving human participants and/or tissue)   - Give the name of the institutional review board or ethics committee that approved the study - Include the approval number and/or a statement indicating approval of this research - Indicate the form of consent obtained (written/oral) or the reason that consent was not obtained (e.g. the data were analyzed anonymously)   Animal Research (involving vertebrate animals, embryos or tissues)   - Provide the name of the Institutional Animal Care and Use Committee (IACUC) or other relevant ethics board that reviewed the study protocol, and indicate whether they approved this research or granted a formal waiver of ethical approval - Include an approval number if one was obtained - If the study involved *non-human primates*, add *additional details* about animal welfare and steps taken to ameliorate suffering - If anesthesia, euthanasia, or any kind of animal sacrifice is part of the study, include briefly which substances and/or methods were applied   Field Research  Include the following details if this study involves the collection of plant, animal, or other materials from a natural setting:   - Field permit number - Name of the institution or relevant body that granted permission |  |
| --- | --- |
|  |  |
| Data Availability  Authors are required to make the data underlying their research findings fully available, without restriction.  If the manuscript reports pilot or preliminary results, the data underlying those results must be made available at the time of publication. A Data Availability | N/A - No results are reported |

*Powered by Editorial Manager® and ProduXion Manager® from Aries Systems Corporation*

| Statement describing where the data can be found is required at submission. Your answers to this question constitute the Data Availability Statement and will be published in the article, if accepted. When legal or ethical requirements prohibit public data sharing, authors must indicate how researchers may obtain access to the data. See the [PLOS Data Policy](https://journals.plos.org/plosone/s/data-availability) and [FAQ](https://journals.plos.org/plosone/s/data-availability#loc-data-policy-faqs) for detailed information. |  |
| --- | --- |
| Important: Stating ‘data available on request from the author’ is not sufficient. If your data are only available upon request, select ‘No’ for the first question and explain your situation in the text box. |  |
| For protocols without pilot or preliminary data, authors are strongly encouraged to state how they plan to share research data from their study when it is completed or published.  If your study includes pilot data, will they be made fully available without restriction? |  |
| Describe where the data may be found in full sentences. If you are copying our sample text, replace any instances of XXX with the appropriate details.   - If no pilot data are reported in the manuscript, please enter the following: *No datasets were generated or analysed during the current study. All relevant data from this study will be made available upon study completion.* - If the manuscript reports pilot data held in a public repository, include URLs, accession numbers or DOIs. If this information will only be available after acceptance, indicate this by ticking the box below. For example: *All XXX files are available from the XXX database (accession number(s) XXX, XXX.).* - If the manuscript reports pilot data contained within the manuscript and/or | No datasets were generated or analysed during the current study. All relevant data from this study will be made available upon study completion. |

*Powered by Editorial Manager® and ProduXion Manager® from Aries Systems Corporation*

| Supporting Information files, enter the following:  *All relevant data are within the manuscript and its Supporting Information files.*   - If neither of these applies but you are able to provide details of access elsewhere, with or without limitations, please do so. For example:   *Data cannot be shared publicly because of [XXX]. Data are available from the XXX Institutional Data Access / Ethics Committee (contact via XXX) for researchers who meet the criteria for access to confidential data.*  *The data underlying the results presented in the study are available from (include the name of the third party and contact information or URL).*   - This text is appropriate if the data are owned by a third party and authors do not have permission to share the data.   For study protocols with data management or sharing plans, authors are encouraged to briefly describe in their Data Availability Statement how data they generate will be made accessible when the study is completed. For example:   - Deidentified research data will be made publicly available when the study is completed and published.   * typeset |  |
| --- | --- |
| Additional data availability information: | Tick here if the URLs/accession numbers/DOIs will be available only after acceptance of the manuscript for publication so that we can ensure their inclusion before publication. |

*Powered by Editorial Manager® and ProduXion Manager® from Aries Systems Corporation*

Manuscript	[Click here to access/download;Manuscript;Manuscript.docx](https://www.editorialmanager.com/pone/download.aspx?id=34523680&guid=1d2dd316-e5af-43c0-9ca2-366936dcb91f&scheme=1)[<https://www.editorialmanager.com/pone/download.aspx?id=34523680&guid=1d2dd316-e5af-43c0-9ca2-366936dcb91f&scheme=1>](https://www.editorialmanager.com/pone/download.aspx?id=34523680&guid=1d2dd316-e5af-43c0-9ca2-366936dcb91f&scheme=1)

## 1 Shared decision making applied to self-management program for

2 **hypertensive patients: A scoping review protocol**

3

4	So Young Yun^1^, Mi Ok Song^2*^

5

6 ^1^Department of Nursing, Nambu University, Gwangju 62771, South Korea,
7 [yunsy@nambu.ac.kr](mailto:yunsy@nambu.ac.kr)
8 ^2^Department of Nursing, Nambu University, Gwangju 62771, South Korea,
9 [coffeesong79@gmail.com](mailto:coffeesong79@gmail.com)

10

11 * Corresponding author
12 Email: [coffeesong79@gmail.com](mailto:coffeesong79@gmail.com)

13

14

15	¶These authors contributed equally to this work.

16	**Abstract**

### 17 Introduction

18 Shared decision making (SDM) improves health outcomes by providing individualized nursing
19 interventions to patients and educating and empowering them to actively participate in health.
20 However, hypertensive patients who require self-management do not actively participate in the
21 SDM process.

### 22 Aims

23 This scoping review aims to investigate the available information on SDM to self- management
24 programs for hypertensive patients.

### 25 Methodology

26 The proposed scoping review protocol will be conducted using the Arksey and O’Malley
27 methodological framework, following the Joanna Briggs Institute’s methodology for scoping
28 reviews. Multiple databases will be searched, including the MEDLINE (PubMed), Embase,
29 Cochrane Library and Google Scholar. Papers’ titles, abstracts, and full text will predominantly
30 be screened by one researcher with a second researcher. Data will be extracted using a
31 customized table developed in collaboration with the research team, and presented in tabular
32 format, arranged thematically. In the scoping review, studies involving the self- management
33 program applied for hypertensive patients with two or more of the components of the
34 intervention are included. And in the SDM process, studies in which healthcare providers
35 provide various options for patients' self- management and set health goals in consideration of
36 patients' preferences and values will be included.

### 37 Registration

38 The scoping review protocol has been registered on the Open Science Framework (OSF)
39 repository Registries (<https://osf.io/rtfvq>; DOI: [10.17605/OSF.IO/D8SPM](https://doi.org/10.17605/OSF.IO/D8SPM)).

40

41	Key word: Decision making, Shared; self-management; hypertension; scoping review

42

# 43 Introduction

44 Shared decision-making (SDM) refers to the process by which at least two participants
45 share information and collaborate to reach an agreement on the preferred treatment of both
46 parties [1]. SDM is a process in which decisions are made in a collaborative manner, where
47 reliable information is provided along with alternative options, and consensus is reached that
48 considers the concerns, values, goals, and preferences of patients and their family [2]. Recently,
49 the patient's participation in decision-making has been emphasized in the process of
50 determining treatment methods, and the informed preferences of patients and their family
51 should play the most important role in the decision-making process [3]. Many organizations
52 around the world are recommending the use of SDM in decision-making in healthcare
53 situations [4,5].
54 Self- management is a process of maintaining health through health promotion activities
55 and disease management [6], and hypertensive patients need to adapt to self- management
56 behaviors. Self-management for hypertensive	patients includes medication, lifestyle
57 management, and regular hospital visits, etc. [7]. Nurses provide self- management support and
58 decision-making support to patients through these self- management interventions and allow
59 patients to participate in the self- management process and obtain information. SDM is also
60 applied to this process, and by encouraging patient participation in the self- management
61 process and providing necessary support to patients, it improves the quality of the decision-
62 making process and treatment implementation, ultimately improving patient outcomes [2]. In
63 the recent middle-range theory of nursing in hypertensive patients, the concept of SDM was
64 presented to the nursing management process for lifestyle changes in hypertensive patients,
65 and problem-solving through mutual goal setting was emphasized [7]. As such, it is necessary
66 to apply SDM to self- management programs for hypertensive patients (SMPHP).
67 SDM strengthens patients' autonomy by providing individualized nursing interventions to
68 patients and taking responsibility for their daily decisions about health problems [8]. In addition,
69 educating and empowering patients to actively participate in their health, improves health
70 outcomes such as compliance with medication [9,10], overall health status, disease knowledge,
71 and improvement in lifestyle [10]. However, there are concerns that hypertensive patients may
72 not actively participate in the SDM process [11,12]. Both healthcare providers and patients
73 experience stress about participating in the SDM process for reasons such as unfamiliarity with
74 SDM [12], problems with old age and health literacy, and differences in knowledge between
75 healthcare providers and patients [13].
76 In this respect, it is necessary to discuss how to effectively apply SDM to SMPHP. To
77 increase the participation of hypertensive patients in decision-making, it is necessary to
78 consider how to form a supportive relationship with medical service providers, motivate
79 patients, and provide accurate and sufficient health information [14]. Regarding the method
80 specifically applied in the SDM process, it would be systematic to consider the three-talk model
81 of SDM as a standard. In the model, the SDM process is explained by dividing it into 'team
82 talk', 'option talk', and 'decision talk' [2].
83 Hypertension causes various cardiovascular diseases, increases premature mortality, and
84 imposes a significant burden on individuals, family, and society. In Korea, the prevalence of
85 hypertension in those aged 19 and older increased from 23.7% in 2010 to 29.0% in 2020, but
86 the control rate was 47.6%, which has not changed significantly over the past 10 years [15]. In
87 this context, the government is implementing a hypertension registration management project,
88 and each public health center is implementing self-management intervention through health
89 education, individual phone counseling, and visiting health services. However, the application
90 of SDM to SMPHP is insufficient [14]. An integrative review that identified the comprehensive
91 process of SDM in healthcare settings [16] and a systematic literature review of the effects of
92 SDM applied to hypertensive patients were conducted [17], but there was no study on the topic
93 of applying SDM to SMPHP.
94 There are currently no scoping review papers summarizing existing research on this topic
95 to help identify evidence and methodological differences. Therefore, the scoping review to
96 contrast and discuss existing studies in this field is meaningful due to differences between
97 previous studies, various methodologies, patient populations, inclusion and exclusion criteria.
98 Due to the heterogeneity and scope of available literature, scoping review are acceptable
99 methodologies that can be used rather than systematic reviews that require a narrower selection
100 of research questions and quality evaluation papers [18].

| 101 | **Aims** |
| --- | --- |
| 102 | The aims of the proposed ScR are: |
| 103 | 1. To summarize the current evidence on the SDM as it relates to SMPHP. |
| 104 | 2. To identify existing gaps to inform future research in this area. |
| 105 |  |
| 106 | **Materials and methods** |

107 The proposed scoping review will be conducted using the Arksey and O’Malley
108 methodological framework [18] and follows the Joana Briggs Instituted (JBI) methodology for
109 scoping reviews [19]. A checklist for the development and reporting of this ScR protocol [20]
110 is included within the supplementary material (S1 Checklist) and a full scoping review will be
111 reported according to PRISMA extension for Scoping Review (PRISMA-ScR) [21].

112

## 113 Stage 1: Identifying the research questions

114 **Research Question**
115 The scoping review question is as follows.
116 1. How have SDM progresses been applied to SMPHP in various healthcare
117 environments?
118 2. What methods were used to actively involve the subject in the SDM process?
119 3. Are there any considerations in the SDM process for the effectiveness of the self-
120 management program?
121 **Types of participants.** Studies involving patients aged 19 years or older with a diagnosis
122 of hypertension will be selected. Hypertensive patients with other comorbidities will be
123 selected, but studies involving secondary hypertensive patients will be excluded.
124 **Concept.** The SMPHP in the study will be selected only if two or more of the components
125 of the intervention (medication, diet, physical activity, BP monitor, smoking cessation, etc.) are
126 included. And in the SDM process, studies in which healthcare providers provide various
127 options for patients' self-management and set health goals in consideration of patients'
128 preferences and values will be included.
129 **Context.** In the scoping review, SMPHP that have been implemented in any healthcare
130 environment, whether in an acute hospital setting or a community primary healthcare setting,
131 will be selected, and the occupational group of interventionists will not be limited.
132 **Type of sources.** In the literature selection process, special research designs will not be
133 restricted, but qualitative studies, protocols, opinion papers, and letters that cannot confirm
134 specific components of a self-management program and the progress of SDM will be excluded.

135

## 136 Stage 2: Identifying relevant studies

137 Multiple databases were searched in January 2023, including the MEDLINE (PubMed),
138 Embase, Cochrane Library and Google Scholar. As recommended by the JBI review methods,
139 a three-step search strategy was employed. In the first step, a limited initial search was
140 performed on the research topic in the databases MEDLINE (PubMed) and Embase. Following
141 initial search, we analyzed the text words contained in the titles and abstracts of retrieved
142 articles and the index terms used to describe the articles. A second search was conducted on
143 MEDLINE (PubMed), Embase, and Cochrane Library using all identified keywords and index
144 terms. A search for gray literature was conducted on Google Scholar. Third, we will search for
145 additional sources by examining the reference list of the finally selected literature. An example
146 search strategy for MEDLINE (PubMed) is presented in the supplementary materials of this
147 protocol (S2 File). There are no restrictions on language or year of publication.

148

## 149 Stage 3: Study selection

150 After the literature search, the identified documents are uploaded using EndNote X8 8.2
151 (Clarivate Analytics, Philadelphia, PA, USA) and duplicates are removed. Titles and abstracts
152 are independently will be reviewed by two reviewers to evaluate inclusion and exclusion
153 criteria, and the literature would be first selected. Two reviewers independently will evaluate
154 the entire contents of the initially selected literature to select the literature for final analysis.
155 Any disagreements that arise during the selection process should be discussed to reach a
156 consensus. The literature search and selection process will be presented in the PRISMA-ScR
157 Flowchart [21]. Before the team begins source selection, a pilot test is conducted with a random
158 sample of 25 titles/abstracts. Screening begins when 75% consent of the team is achieved.

159

## 160 Stage 4: Charting the data

161 **Data extraction.** Data will be extracted using a data extraction tool developed by the
162 researcher based on the data extraction part of the JBI scoping review [18]. The components of
163 the self-management program and the process of SDM will be extracted along with the general
164 characteristics of the literature. The SDM process is divided into team talk, option talk, and
165 decision talk [2] to extract data. The details of the extraction tool are shown in Table 1. Before
166 data extraction, a pilot test will be conducted using the extraction tool for the two selected
167 literature, and then it was confirmed that all relevant results were extracted. The data extraction
168 tool can be modified during the data extraction process and will be written in such a way that
169 one reviewer extracts independently and the other reviewer verifies it. Any disagreement
170 between reviewers will be resolved through discussion during data extraction process.

### 171 Table 1. Data extraction tool

| Categories | Sub-categories | Details |
| --- | --- | --- |
| General characteristics | Authors  Year of publication |  |

|  | Country of origin Study design |  |
| --- | --- | --- |
| Self-management program | Setting Participants  Blood pressure measurement Component |  |
| How shared decision making was applied | Three-talk model | Team talk |
|  |  | Option talk |
|  |  | Decision talk |
|  | Decision aid |  |
|  | Evaluation |  |
| Outcomes |  |  |

182

| 172 |  |
| --- | --- |
| 173 | **Stage 5: Collating, summarizing, and reporting the results** |
| 174 | **Data analysis and presentation.** Data analysis will be presented by analyzing the results |
| 175 | produced by the extraction tool. A descriptive analysis of the general characteristics of the |
| 176 | literature and an analysis of research questions will be conducted. Analysis based on the theory |
| 177 | of the SDM process will be used when applying the SMPHP in the future, and the three-talk |
| 178 | model and implementation of the SDM process will be compared. Quantitative data will use |
| 179 | descriptive statistics, and descriptive summaries and tabular results will be used to characterize |
| 180 | SMPHP. The results of this study will reveal strategies for applying SDM when planning a |
| 181 | SMPHP. |
| 183 | **Ethical Consideration** |
| 184 | This study will begin analysis after receiving approval of the application for exemption from |
| 185 | deliberation by the Institutional Review Board of Nambu University (IRB #1041478-2021- |
| 186 | HR-035). |

187

## 188 Strengths and limitations of this study

189 • The review will be one of the first study to examine the SDM process of SMPHP.
190 • Exploring this issue has the potential to highlight the areas that impact on the intervention of
191 SMPHP.
192 • It would also inform healthcare providers about the challenges and the needs for SDM of
193 SMPHP so that support and resources are directed to increase effectiveness.

194

## 195 Registration

196 This project has been created for the Scoping Review on the OSF repository and the protocol
197 and search strategies uploaded. The project has also been registered with the OSF Registries.
198 • OSF project: <https://osf.io/rtfvq>
199 • DOI: [10.17605/OSF.IO/D8SPM](https://doi.org/10.17605/OSF.IO/D8SPM)

200

# 201 Supporting information

202 S1 Checklist. PRISMA-P 2015 Checklist for reporting scoping review protocol. (DOCX)
203 S2 File. Sample search strategy for MEDLINE (PubMed). (DOCX)

204	**Acknowledgments**

205 We would like to thank those who conducted the study for SDM processes implemented within
206 SMPHP so that this study could be conducted. This study was supported by research funds
207 from Nambu University, 2022. But the funders had and will not have a role in study process.

208

# 209 Author Contributions

210 Conceptualization: So young Yun, Mi Ok Song.
211 Data curation: So young Yun, Mi Ok Song.
212 Methodology: So young Yun, Mi Ok Song.
213 Supervision: So young Yun.
214 Writing – original draft: So young Yun, Mi Ok Song.
215 Writing – review & editing: Mi Ok Song.

216

217	**References**

218 1. Charles C, Gafni A, Whelan T. Shared decision-making in the medical encounter: what does
219 it mean? (or it takes at least two to tango). Social Science & Medicine. 1997;44(5):681-692.
220 <https://doi.org/10.1016/S0277-9536(96)00221-3>
221 2. Elwyn G, Durand MA, Song J, Aarts J, Barr PJ, Berger Z, et al. A three-talk model for shared
222 decision	making:	multistage	consultation	process.	BMJ.	2017;359:j4891.
223 <https://doi.org/10.1136/bmj.j4891>
224 3. Spatz ES, Krumholz HM, Moulton BW. Prime time for shared decision making. *JAMA*
225 2017;317(13):1309-1310. <https://doi.org/10.1001/jama.2017.0616>
226 4. National Institute for health and care Excellence. Shared decision making [Internet]. London:
227 NICE; [cited 2021 May 3]. Available from: [https://www.nice.org.uk/about/what-we-do/our-](https://www.nice.org.uk/about/what-we-do/our-programmes/nice-guidance/nice-guidelines/shared-decision-making)
228 [programmes/nice-guidance/nice-guidelines/shared-decision-making](https://www.nice.org.uk/about/what-we-do/our-programmes/nice-guidance/nice-guidelines/shared-decision-making)
229 5. Kon AA. The shared decision-making continuum. JAMA. 2010;304(8):903–904.
230 <https://doi.org/10.1001/jama.2010.1208>
231 6. Riegel B, Jaarsma T, Strömberg A. A middle-range theory of self-care of chronic illness.
232 Advances	in	Nursing	Science.	2012;35(3):194-204.
233 <https://doi.org/10.1097/ANS.0b013e318261b1ba>
234 7. Drevenhorn E. A proposed middle-range theory of nursing in hypertension care.
235 International	Journal	of	Hypertension.	2018;2018:2858253.
236 <https://doi.org/10.1155/2018/2858253>
237 8. Schoenthaler A, Rosenthal DM, Butler M, Jacobowitz L. Medication adherence
238 improvement similar for shared decision-making preference or longer patient-provider
239 relationship. The Journal of the American Board of Family Medicine. 2018;31(5):752-760.
240 <https://doi.org/10.3122/jabfm.2018.05.180009>
241 9. Ben-Zacharia A, Adamson M, Boyd A, Hardeman P, Smrtka J, Walker B, et al. Impact of
242 shared decision making on disease-modifying drug adherence in multiple sclerosis.
243 International	Journal	of	MS	Care.	2018;20(6):287-297.	[https://doi.org/10.7224/1537-](https://doi.org/10.7224/1537-2073.2017-070)
244 [2073.2017-070](https://doi.org/10.7224/1537-2073.2017-070)
245 10. Granados-Santiago M, Valenza MC, López-López L, Prados-Román E, Rodríguez-Torres
246 J, Cabrera-Martos I. Shared decision-making and patient engagement program during acute
247 exacerbation of COPD hospitalization: A randomized control trial. Patient Education and
248 Counseling. 2020;103(4):702-708. <https://doi.org/10.1016/j.pec.2019.12.004>
249 11. Nørgaard B, Titlestad SB, Marcussen M. Shared decision-making in general practice from
250 a patient perspective. A cross-sectional survey. Scandinavian Journal of Primary Health Care.
251 2022 ;28:1-6. <https://doi.org/110.1080/02813432.2022.2069700>
252 12. Johnson R, Turner K, Feder G, Cramer H. Shared decision making in consultations for
253 hypertension: Qualitative study in general practice. Health Expect. 2021;24(3):917-929.
254 <https://doi.org/10.1111/hex.13234>
255 13. Backman WD, Levine SA, Wenger NK, Harold JG. Shared decision-making for older
256 adults	with	cardiovascular	disease.	Clinical	Cardiology.	2020;43(2):196-204.
257 <https://doi.org/10.1002/clc.23267>
258 14. Lee JH. Experience of patients’ having hypertension and diabetes participation in health
259 care. The Journal of the Korea Contents Association. 2021;21(3):552-560.
260 <https://doi.org/10.5392/JKCA.2021.21.03.552>
261 15. Statistics Korea. 2020 Annual report on the causes of death statistics [Internet]. Seoul:
262 Statistics	Korea;	2020	[cited	2021	August	4].	Available	from:
263 <https://kostat.go.kr/portal/korea/kor_nw/1/1/index.board?bmode=read&aSeq=385219>
264 16. Truglio-Londrigan M, Slyer JT. Shared decision-making for nursing practice: an
265 integrative	review.	The	Open	Nursing	Journal.	2018;12:1-14.
266 <https://doi.org/10.2174/187443460181201000>
267 17. Johnson RA, Huntley A, Hughes RA, Cramer H, Turner KM, Perkins B, et al.
268 Interventions to support shared decision making for hypertension: a systematic review of
269 controlled	studies.	Health	Expectations.	2018;21(6):1191-1207.
270 <https://doi.org/10.1111/hex.12826>
271 18. Arksey H, O’Malley L. Scoping studies: Towards a methodological framework.
272 International	Journal	of	Social	Research	Methodology.	2005;8(1):19–32.
273 <https://psycnet.apa.org/doi/10.1080/1364557032000119616>
274 19. Peters MDJ, Godfrey C, McInerney P, Khalil H, Larsen P, Marnie C, et al. Best practice
275 guidance and reporting items for the development of scoping review protocols. JBI
276 Evidence Synthesis. 2022;20(4):953-968. <https://doi.org/10.11124/JBIES-21-00242>
277 20. Moher D, Shamseer L, Clarke M, Ghersi D, Liberati A, Petticrew M, et al. Preferred
278 reporting items for systematic review and meta-analysis protocols (PRISMA-P) 2015
279 statement. Systematic Reviews. 2015;4(1):1–9. <https://doi.org/10.1186/2046-4053-4-1>
280 21. Tricco AC, Lillie E, Zarin W, O’Brien KK, Colquhoun H, Levac D, et al. PRISMA
281 extension for scoping reviews (PRISMA-ScR): checklist and explanation. Annals of

282

283

284

285

Internal Medicine 2018;169(7): 467–473. <https://doi.org/10.7326/M18-0850>

checklist

[Click here to access/download](https://www.editorialmanager.com/pone/download.aspx?id=34222861&guid=05830973-a6a8-45e7-ba02-d804eedeae62&scheme=1)

[Supporting Information S1. Checklist.docx](https://www.editorialmanager.com/pone/download.aspx?id=34222861&guid=05830973-a6a8-45e7-ba02-d804eedeae62&scheme=1)

search strategy

[Click here to access/download](https://www.editorialmanager.com/pone/download.aspx?id=34222801&guid=674d1aa1-74b7-4fbc-8868-91a534de55a9&scheme=1)

[Supporting Information](https://www.editorialmanager.com/pone/download.aspx?id=34222801&guid=674d1aa1-74b7-4fbc-8868-91a534de55a9&scheme=1)

[S2. Search Strategy for MEDLINE (PubMed).docx](https://www.editorialmanager.com/pone/download.aspx?id=34222801&guid=674d1aa1-74b7-4fbc-8868-91a534de55a9&scheme=1)

IRB approval letter

[Click here to access/download](https://www.editorialmanager.com/pone/download.aspx?id=34340963&guid=31ab4157-6673-4baf-a4ca-3b233a7e8363&scheme=1)

[Supporting Information IRB(English Version).pdf](https://www.editorialmanager.com/pone/download.aspx?id=34340963&guid=31ab4157-6673-4baf-a4ca-3b233a7e8363&scheme=1)
