# Supplementary material for: Shared decision making applied to self-management program for hypertensive patients: A scoping review protocol
Source: PLoS One. 2024 Nov 21;19(11):e0309593. doi: 10.1371/journal.pone.0309593 (PMC11581201; doi:10.1371/journal.pone.0309593)
Supplement: Attachment — Submitted filename: Response to Reviewer.docx [file pone.0309593.s004.docx]

June. 16. 2024

Dear Editor and Reviewer:

On behalf of my-co-authors, I thank you and the reviewers for your thoughtful comments and recommendation. We have made the following changes to the manuscript to address your concerns. The revisions are shown in Red in the text.

Response to Editor`s Comments

#1. Please ensure that your manuscript meets PLOS ONE's style requirements, including those for file naming.

=> According to the editor's opinion, manuscript formatting, Title page and file naming have been revised.

#2. Thank you for stating the following financial disclosure:

"This study was supported by research funds from Nambu University, 2022"

Please state what role the funders took in the study. If the funders had no role, please state: "The funders had no role in study design, data collection and analysis, decision to publish, or preparation of the manuscript." If this statement is not correct you must amend it as needed. Please include this amended Role of Funder statement in your cover letter; we will change the online submission form on your behalf.

=> According to the editor's opinion, Authors stated what role the funders took in the study. It is state in the cover letter that "The funders had no role in study design, data collection and analysis, decision to publish, or preparation of the manuscript."

#3. Please provide a complete Data Availability Statement in the submission form, ensuring you include all necessary access information or a reason for why you are unable to make your data freely accessible. If your research concerns only data provided within your submission, please write "All data are in the manuscript and/or supporting information files" as your Data Availability Statement.

=> According to the editor's opinion, it was stated that " No datasets were generated or analyzed during the current study. All relevant data from this study will be made available upon study completion." for Data Availability Statement in the submission form.

#4. When completing the data availability statement of the submission form, you indicated that you will make your data available on acceptance. We strongly recommend all authors decide on a data sharing plan before acceptance, as the process can be lengthy and hold up publication timelines. Please note that, though access restrictions are acceptable now, your entire data will need to be made freely accessible if your manuscript is accepted for publication. This policy applies to all data except where public deposition would breach compliance with the protocol approved by your research ethics board. If you are unable to adhere to our open data policy, please kindly revise your statement to explain your reasoning and we will seek the editor's input on an exemption. Please be assured that, once you have provided your new statement, the assessment of your exemption will not hold up the peer review process.

=> According to the editor's opinion, all authors decided on a data sharing plan before acceptance and completed the data availability statement of the submission form

Response to Reviewer #2`s Comments

#1. You indicate that google Scholar is a database where you are going to carry out your study, I must point out that google scholar is not a scientific database, but a generalist search engine. Furthermore, it would be appropriate to describe in a clearer way the process of search and selection of documents to be included in the study. After reading stage 2, it is not easy to really understand the meaning of the first search and even the use of Google Schola for gray literature search.

=> According to the reviewer's opinion, the literature search process had been revised and suggested in line 128~136 of the manuscript.

Multiple databases were searched in January 2023, including the MEDLINE (PubMed), Embase, Cochrane Library. Following JBI review methods, a three-step search strategy was employed. Initially, a limited search was conducted on the research topic in MEDLINE (PubMed) and Embase database. Subsequently, the text words from titles and abstracts of retrieved articles, along with index terms used to describe the articles, were analyzed. A second search was conducted on MEDLINE (PubMed), Embase, and Cochrane Library using all identified keywords and index terms. Additionally, a search for gray literature was conducted on Google Scholar. Thirdly, additional sources will be searched by examining the reference list of the finally selected literature.

#2 Finally, regarding the document selection criteria, it is necessary to specify in greater detail the elements that will help in the selection of documents.

=> According to the editor's opinion, the document selection criteria were revised at lines 149-155 and exclusion criteria were presented in detail.

Inclusion criteria will comprise adults (aged 19 years and above) diagnosed with Hypertension (HTN), and a self-management intervention encompassing a minimum of two or more intervention components (e.g., education, physical activity, pharmacotherapy, or lifestyle modification). Exclusion criteria will entail secondary and tertiary data studies, abstracts, or protocol-only papers, and inaccessible full texts. SDM intervention is expected to entail an iterative process whereby healthcare providers and participants collaboratively establish goals. Studies where the participant assumes the role of the healthcare provider will be excluded.
